# Long-term Recordings of Arcuate Nucleus Kisspeptin Neurons Across the Mouse Estrous Cycle

**DOI:** 10.1210/endocr/bqae009

**Published:** 2024-01-27

**Authors:** Szilvia Vas, Ellen Wall, Ziyue Zhou, Lajos Kalmar, Su Young Han, Allan E Herbison

**Affiliations:** Department of Physiology, Development and Neuroscience, University of Cambridge, Cambridge CB2 3EG, UK; Department of Physiology, Development and Neuroscience, University of Cambridge, Cambridge CB2 3EG, UK; Department of Physiology, Development and Neuroscience, University of Cambridge, Cambridge CB2 3EG, UK; Department of Toxicology, University of Cambridge, Cambridge CB2 1QR, UK; Department of Physiology, Development and Neuroscience, University of Cambridge, Cambridge CB2 3EG, UK; Department of Physiology, Development and Neuroscience, University of Cambridge, Cambridge CB2 3EG, UK

**Keywords:** kisspeptin, GnRH pulse generator, estrous cycle, k-means clustering, arcuate nucleus, luteinizing hormone, pulsatility

## Abstract

The arcuate nucleus kisspeptin (ARN^KISS^) neurons represent the GnRH pulse generator that likely drives pulsatile gonadotropin secretion in all mammals. Using an improved GCaMP fiber photometry system enabling long-term continuous recordings, we aimed to establish a definitive profile of ARN^KISS^ neuronal activity across the murine estrous cycle. As noted previously, a substantial reduction in the frequency of ARN^KISS^ neuron synchronization events (SEs) occurs on late proestrus and extends into estrus. The SE amplitude remains constant throughout the cycle. During metestrus, we unexpectedly detected many multipeak SEs where many SEs occurred rapidly, within 160 seconds of each other. By applying a machine learning-based, k-means clustering analysis, we were further able to detect substantial within-stage variability in the patterns of pulse generator activity. Estrous cycle-dependent changes in SE activity occurred around the time of lights on and off. We also find that a mild stressor such as vaginal lavage reduces ARN^KISS^ neuron SE frequency for up to 3 hours. These observations provide a comprehensive account of ARN^KISS^ neuron activity across the estrous cycle, highlight a new pattern of multipeak SE activity, and introduce a new k-means clustering approach for analyzing ARN^KISS^ neuron population behavior.

A delicate interplay between the brain and ovaries is responsible for orchestrating the fluctuating reproductive cycles of female mammals. Principally, this involves the feedback actions of estradiol and progesterone on the pituitary gonadotrophs and the neural networks controlling the pulsatile and surge modes of GnRH secretion into the pituitary portal circulation (1-3). The resulting dynamic profiles of circulating LH and FSH across the estrous cycle are well described in many species (4).

A discrete population of kisspeptin neurons located in the arcuate nucleus (ARN) is now considered to be the pulse generator driving the episodic secretion of gonadotrophic hormones in mammals (5-7). The other kisspeptin-expressing neuron population located in rostral periventricular area of the third ventricle is believed to drive the preovulatory surge of LH (8, 9).

Recent GCaMP fiber photometry data indicate that the ARN kisspeptin (ARN^KISS^) neurons become synchronously active for approximately 1 minute every hour throughout much of the mouse estrous cycle, with a dramatic reduction in synchronization events (SEs) during estrus (10, 11). Although informative, those studies were limited in that estrous cycle evaluations of pulse generator activity were made primarily from short recording sessions during the morning and afternoon.

We have recently made improvements to GCaMP fiber photometry methodologies for ARN^KISS^ neurons that enable long-term recordings to be made for up to 96 hours. This has allowed us to make continuous, undisturbed recordings of ARN^KISS^ neuron population SEs across multiple days of the estrous cycle.

We provide here a detailed characterization of pulse generator activity throughout the estrous cycle and report unexpected new patterns of activity of the GnRH pulse generator across the estrous cycle with, in particular, the discovery of multipeak SEs (mpSEs) occurring predominantly during metestrus. Furthermore, using unsupervised k-means clustering, we show that distinct elements of ARN^KISS^ neuron SE behavior can be used to independently describe the estrous cycle-dependent alterations in the activity of the pulse generator.

## Methods

### Animals

To express GCaMP6s selectively in kisspeptin neurons, 129S6Sv/Ev C57BL/6 *Kiss1^Cre/+^*mice (12) were crossed with the Ai162 (TIT2L-GC6s-ICL-tTA2)-D Cre-dependent GCaMP6s line (JAX stock #031562) (13) to generate *Kiss1^Cre/+^, Ai162^+/+^* mice (N = 11) (14). Some recordings were also undertaken in mice (N = 7) additionally carrying the Cas9 reporter allele (Gt(ROSA)26Sor^tm1(CAG-cas9*,−EGFP)Fezh^/J). Recordings were made from ages 10 to 39 weeks. Before surgery, mice were group-housed in conventional cages with environmental enrichment under controlled laboratory conditions (22 ± 2 °C, 12/12-hour light-dark cycle with lights on at 7 Am) with ad libitum access to food (RM3, Vivo Bio Tech, UK) and water. Following surgery, mice were single housed until the end of the study. All animals used in this study were controls for other studies in the laboratory, so that no animal was implanted solely for this project. All animal experimental protocols were approved by the University of Cambridge, UK (P174441DE).

### Stereotaxic Surgery

Female mice were pretreated subcutaneously with meloxicam (5 mg/kg), buprenorphine (0.05 mg/kg), and dexamethasone (10 mg/kg), anesthetized with isoflurane (1%-2%, 1 L/min) and placed in a stereotaxic frame. An optical fiber (400-µm diameter, Doric Lenses, Quebec, Canada) was positioned directly above the mid-caudal ARN (2.0-mm posterior to Bregma, 0.35 mm lateral to midline, 5.93-mm deep from brain surface). For postoperative pain relief, meloxicam (5 mg/kg) was administered orally for up to 2 days. Following surgery, postoperative monitoring was provided for 5 days. Daily behavioral training and habituation to the recording conditions started at least 7 days after surgery.

### GCaMP6 Fiber Photometry

Fiber photometry data were collected using a custom-built fiber photometry rig including optical components from Doric Lenses (Quebec, Canada) and a data acquisition board from National Instrument (Texas, USA) based on a previous design (14). Calcium-dependent (blue, 465-490-nm) and non-calcium-dependent (violet, 405-nm) excitation lights were sinusoidally modulated at 531 and 211 Hz, respectively, and were focused onto a 400-μm diameter fiber optic connected to the mouse. The emitted fluorescence was collected by the same fiber and conveyed through a 500- to 550-nm emission filter, and then focused onto a fluorescence detector. Fluorescence signals were detected at 10 Hz using a scheduled mode (5 seconds on, 15 seconds off) or, by using a continuous mode of light emission for shorter recordings to characterize the profile of the individual SEs. The 2 GCaMP6s emissions were recovered by demodulating the 465 through 490-nm and the 405-nm signals. Mice were connected to the fiber photometry system using a fiber optic patch cord. Photometry recordings were started on any stage of the estrous cycle between 9 and 11 Am and continued for at least 24 hours. The numbers of mice with continuous duration recording sessions were 24 hours (N = 24), 48 hours (N = 5), 72 hours (N = 3) and 96 hours (N = 7). All mice included in the study contributed at least 2 recording sessions and estrous cycle data were generated from a single recording from each mouse. Continuous 96-hour recordings were begun at 10 or 11 Am in proestrus or estrous in 5 *Kiss1*-GCaMP6 animals.

### Estrous Cycle Determination

The estrous cycles of each mouse were characterized by daily vaginal smearing over at least 3 weeks before starting the experiment and only regularly cycling mice used. Stages of the estrous cycle were determined at the start of the recording (9-11 Am) and on subsequent days by vaginal lavage and cytology. Cells were collected from the vaginal canal by using 5 μL of phosphate buffer flushed into the vagina by a micropipette. The tip of the pipette was carefully inserted at a depth of approximately 1 to 2 mm. A small drop of the sample was placed on a slide and stained with Wright's Giemsa for the identification of the different cell types. Stages of the estrous cycle were determined based on the predominance of nucleated or cornified epithelial cells, as well as leukocytes, as characteristic of proestrus, estrus, and metestrus/diestrus, respectively (15).

### Data Analysis

For analysis, recordings from 18 mice were grouped into 6-hour blocks according to the time of lights off (7 Pm) so that the time intervals for each day of the estrous cycle were 7 Am to 1 Pm, 1 Pm to 7 Pm, 7 Pm to 1 Am, 1 Am to 7 Am, and 7 Am to 1 Pm again. Because connecting to the recording system and performing vaginal smearing is sufficiently stressful for mice to impact pulse generator activity (see Results), all data in the first time block (7 Am-1 Pm) when this occurred were omitted from the detailed (6-hour time resolution) analyses. Instead, data for the 7 Am to 1 Pm time block was obtained from the end of an appropriate stage period of recording up until the time of vaginal smearing in the late morning. As such, the 7 Am to 1 Pm bin could contain less than 6 hours of data. All other time blocks had full 6-hour data sets.

Signal processing was performed in Matlab (MathWorks, Inc.). Briefly, non-calcium-dependent background emissions were subtracted from the calcium-dependent emissions and baseline shift corrected by the “msbackadj” Matlab function (by applying a 900-second window size). For detecting SEs, the “findpeaks” Matlab function was used. Peaks in fluorescence larger than 1/3 of the highest peak over each 24-hour recording period were considered to represent an SE. Peaks with >30 seconds of time difference were counted as separate peaks. Because recording conditions influence the amplitude of SEs even between recordings undertaken on the same mouse, SE amplitude was quantified only from continuous recordings (>72 hours) and normalized to the highest amplitude of the whole recording session.

Recordings were segmented into 24-hour periods and a custom MATLAB code used to extract features of the population activity of the ARN^KISS^ neurons to determine: (1) number of SEs within a given period; (2) presence and amount of different type of SEs (single and multipeak SEs); (3) inter-SE intervals, variation in the inter-SE intervals (reflecting regular or irregular patterns of activity); and (4) shape of the SEs (height, variation in height, width of the amplitude at is half height).

### Statistical Analysis

All statistical analyses were performed using GraphPad Prism 9 software. Values represent mean ± standard error of the mean. N represents the number of mice. The level of significance was defined at α = .05. Where the distribution of the dataset did not pass the Shapiro-Wilk normality test, nonparametric tests were applied. Outliers were identified with the ROUT method (GraphPad Prism 9) based on the false discovery rate by fitting a curve with nonlinear regression at α = .01.

The frequency of SEs recorded from ARN^KISS^ neurons across cycle stages was analyzed by 2-way repeated measures (RM) ANOVA (repeated factor: time). The effect of vaginal smearing on the number of SEs was analyzed by 1-way RM ANOVA (repeated factor: time bins). For post hoc comparisons after ANOVA, the Holm-Sidak multiple comparison test was used. The variability (SD) of inter-SE intervals and parameters characterizing mpSEs (frequency and number of peaks) over 24 hours were analyzed by Kruskal-Wallis test followed by Dunn multiple comparisons.

### K-means Clustering

We performed unsupervised k-means clustering to examine if any specific feature or group of SE characteristics can describe the estrous cycle-dependent alterations in the activity of the ARN^KISS^ neuron population without labeling data according to the vaginal cytology. First, we eliminated those parameters that had a high correlation with a retained feature (eg, mean of inter-SE intervals) or a known external factor (eg, recording system, animal). This filtering step led to the exclusion of most of the features describing the SE peak shape because these parameters highly correlated with individual animals and recording systems. Then, we fragmented the 24-hour recordings by using a 7-hour window size with 1-hour sliding steps to extract highly overlapping regions of activity from the SE pattern data. In each 24-hour segment of the recordings, the first and the last 3 hours of each day were extrapolated from the assignment of the fourth and 21st hours, respectively.

Five SE parameters were identified as being sufficient to describe the full behavior of the ARN^KISS^ neuron synchronization patterns across the cycle. These included: (1) number of SEs, where mpSEs were counted as a single SE; (2) SD of inter-SE intervals; (3) number of mpSEs; (4) average number of peaks within mpSEs; and (5) average duration of mpSEs (Supplementary File) (16). Before performing k-means clustering, these parameter values were linearly rescaled to values between 0 and 1. To perform k-means clustering on the data set, we used RapidMiner Studio's (https://rapidminer.com, educational license) built-in module (k = 5, number of runs with random initiations = 100, measure types = “NumericalMeasures,” numerical measure = “EuclideanDistance,” maximum optimization steps = 100). Cluster centroid data and cluster labels (to each 7-hour window) were exported from RapidMiner Studio and were visualized in R (using “ggplot2,” “readr,” and “fmsb” libraries).

## Results

### Estrous Cycle-dependent Activity Pattern of the ARN^KISS^ Neurons

**Figure 1.**
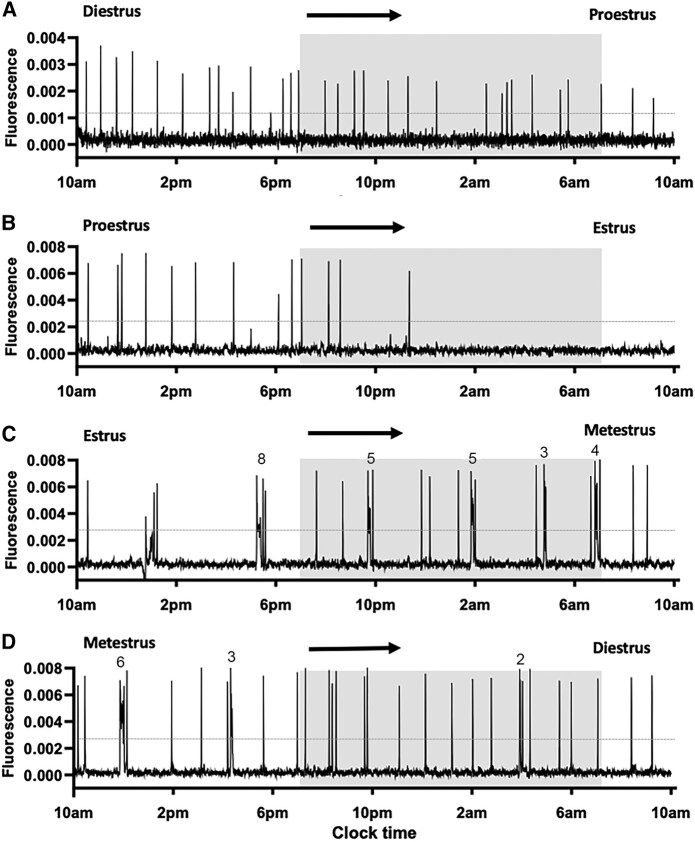
ARN^KISS^ neuron pulse generator activity across the estrus cycle. (A-D) Representative 24-hour GCaMP photometry traces recorded from the same female mouse transitioning from diestrus to proestrus (A), proestrus to estrus (B), estrus to metestrus (C), and from metestrus to diestrus (D). The shaded area indicates the period of lights off (from 7 Pm to 7 Am). The dotted line shows the threshold for detecting synchronization events. Note the emergence of the multipeak synchronization events (mpSEs) in the estrus and metestrus stages (C, D). Numbers above the traces indicate the number of individual peaks in each mpSE. Estrous stage was determined at the beginning and at the end of each recording. Each stage of the estrous cycle was defined as beginning at midnight.

**Figure 2.**
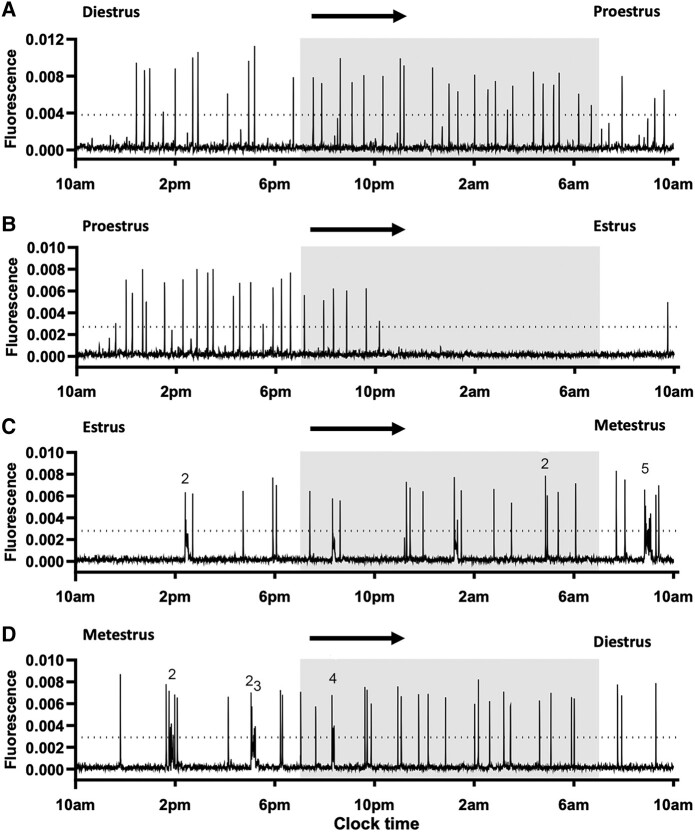
ARN^KISS^ neuron pulse generator activity across the estrus cycle. (A-D) Representative 24-hour GCaMP photometry traces recorded from a different female mouse transitioning from diestrus to proestrus (A), proestrus to estrus (B), estrus to metestrus (C), and from metestrus to diestrus (D). The shaded area indicates the period of lights off (from 7 Pm to 7 Am). The dotted line shows the threshold of detecting synchronization events. Note the emergence of the multipeak synchronization events (mpSEs) in the estrus and metestrus stages (C, D). Numbers above the traces indicate the number of individual peaks in each mpSE. Estrous stage was determined at the beginning and at the end of each recording. Each stage of the estrous cycle was defined as beginning at midnight.

As observed previously (10), we find that abrupt ∼1-minute duration increases in GCaMP activity, representing an SE, occur across the estrous cycle with a dramatic slowing on the evening of proestrus into estrus (Figs. 1 and 2 and Supplementary File) (16). Additionally, however, we observe the occurrence of distinct mpSEs beginning during the estrus to metestrus transition and slowly becoming less frequent into diestrus (Fig. 2C and 2D). No differences were detected between Kiss1-Cre,Ai162 (N = 11) and Kiss1-Cre,Ai162,Cas9 (N = 7) mice in the occurrence of mpSEs across the estrous cycle.

**Figure 3.**
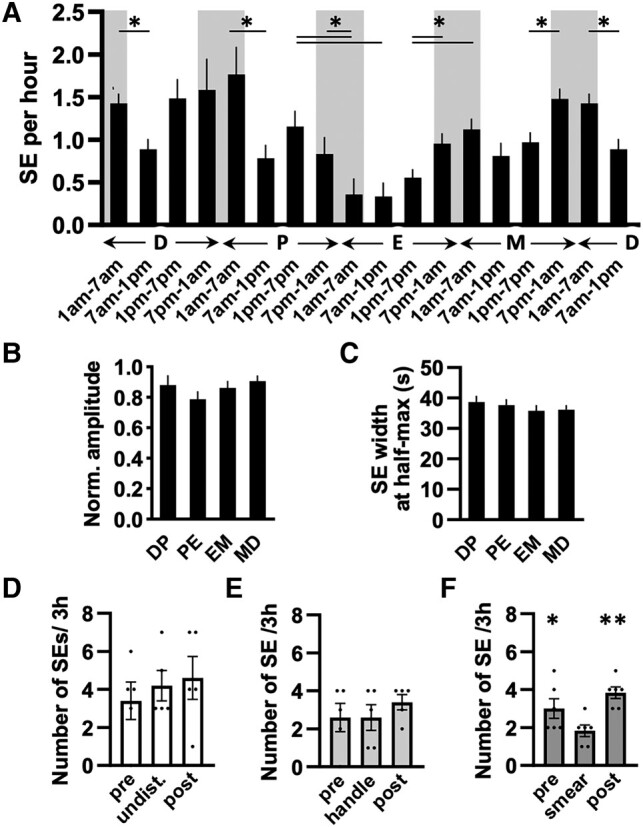
Bar graphs showing the frequency and other features of synchronization events (SEs) of the ARN^KISS^ neurons across the estrous cycle. (A) Average number of SEs per hour plotted in 6-hour bins (N = 10-16 mice/stage) over 24 hours (N = 10, 14, 15, and 16 recordings started in D, P, E, and M, respectively). The “lights off” period (from 7 Pm to 7 Am) is indicated by shading. *P < .05 shows significant difference vs other 6-hour periods indicated by lines above the bars. Abbreviations: D, diestrus; E, estrus; M, metestrus; P, proestrus. Note that the 1 Am to 7 Am and 7 Am to 1 Pm bars in diestrus are double plotted. (B) Amplitude of SEs normalized to the highest SE amplitude value across all recordings from the same mouse over the estrous cycle (>72 hours, N = 4-10 mice/stage). (C) Width of SEs measured at the half-maximum of the amplitude (N = 10-16 mice/stage). (B, C) The analysis was performed in DP, PE, EM, and MD, meaning 24-hour recordings during which mice were transitioning from proestrus to estrus (PE), estrus to metestrus (EM), metestrus to diestrus (MD), and diestrus to proestrus (DP). (A-C) All recordings were started between 9 and 11 Am. (D-F) Changes in SE frequency over three 3-hour time blocks in diestrous mice that were undisturbed (N = 5); (D), following “sham lavage” handling (N = 5); (E) or vaginal lavage (N = 6); (F) occurring at the start of the second 3-hour period, respectively. The 3-hour time bins started at 7 Am, 10 Am, and 1 Pm. **P < .01 and *P < .05. Data are represented as mean ± SEM. For post hoc analysis, Holm-Sidak multiple comparison test was used.

We first assessed SE frequency across the estrous cycle by considering each mpSE as a single event. Here, following the traditional concept, each stage of the estrous cycle was defined as beginning at midnight (on the day before the vaginal cytology was performed). This revealed marked estrous cycle- and time-dependent variations (2-way RM ANOVA: estrous stage: F(3,51) = 4.88, *P* = .0046; time: F(2.42, 123.6) = 17.86, *P* < .0001; time × stage interaction: F(9,153) = 8.08, *P* < .0001) (Fig. 3A). Synchronization event frequency was relatively stable at 1.0 to 1.5 SEs/hour across diestrus and proestrus with the exception of a slowing at diestrus 7 Am through 1 Pm compared with earlier diestrus times (Fig. 3A). Events then slowed substantially during estrous to a nadir of ∼0.35 SE/hour (Fig. 3A) followed by a return to 1.0 through 1.5 SEs/hour during metestrus (Fig. 3A). The amplitude and duration of SEs did not change across the estrous cycle (Fig. 3B and 3C).

The time of “lights off” and “lights on” was found to be associated with cycle stage-dependent changes in pulse generator activity. Overall, the frequency of SEs during the 6-hour periods before and after the light switch was significantly different (Holm-Sidak post hoc comparison: *P* < .01) (Fig. 3A). The frequency of SEs significantly increases at this time in both estrous (72%, *P* = .0016) and metestrous (53%, *P* = .0001) stages but is not changed at diestrus and proestrus (Fig. 3A). The “lights on” had the opposite effect of decreasing SE frequency in diestrus (38%, *P* < .0001) and proestrus (55%, *P* = .007) but not in estrus and metestrus.

To assess the impact of handling and vaginal lavage on pulse generator activity, SE frequency was assessed in three 3-hour bins with either nothing (N = 5), handling to simulate vaginal lavage but without any lavage (N = 5), and vaginal lavage (N = 6) being undertaken at the start of the second 3-hour period. Mice remained connected to the recording apparatus throughout. Pulse generator activity in mice left undisturbed or experiencing the “sham” vaginal lavage was unchanged throughout the 9-hour period (Fig. 3D and 3E). However, a significant 39% decrease in SE frequency occurred in the 3-hour period during which the vaginal lavage occurred (F(1.164, 5.821) = 10.28, *P* = .0175) with pulse generator activity returning to normal in the last 3-hour interval (Fig. 3F).

### ARN^KISS^ Neurons Show Multipeak Synchronization Events

**Figure 4.**
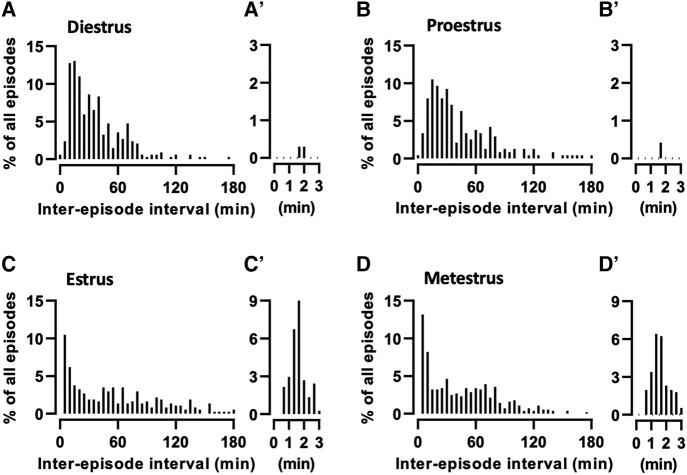
Histograms showing the distribution of the inter-SE intervals as the percentage of all inter-SE intervals in the given estrous stage in 5-minute time bins between 0 and 180 minutes. (A-A′) The 24-hour recordings during which mice were transitioning from diestrus to proestrus (n = 337 events, N = 10 mice), (B-B′) proestrus to estrus (n = 238, N = 14), (C-C′) estrus to metestrus (n = 372, N = 15), and (D-D′) and from metestrus to diestrus stages (n = 562, N = 16). (A′, B′, C′, and D′) Histograms plotting all inter-SE intervals less than 3 minutes for each stage in 20-second time bins. Note that short inter-SE intervals were detected almost exclusively in estrous and metestrous stages (C′ and D′). All photometry recordings were started between 9 and 11 Am. Estrous stage was determined at the beginning and at the end of each recording.

To help define the characteristics of the mpSEs, we first assessed inter-SE intervals at each of the estrous cycle stages (Fig. 4). During diestrus and proestrus, the inter-interval histogram showed intervals to be clustered around 15 to 40 minutes with a relatively normal distribution and right-sided tail (Fig. 4A and 4B). In contrast, during both estrus and metestrus, a high frequency of SE event intervals occurred in the first 5-minute bin (Fig. 4C and 4D) and closer inspection identified a cluster of intervals around 1.5 minutes (Fig. 4C′ and 4D′). These intervals all corresponded to events occurring within mpSEs and have inter-SE interval times of 45 to 160 seconds (Fig. 4C′ and 4D′). As such, mpSEs were defined as times when SEs occurred <160 seconds apart.

The inter-SE interval histogram analysis (Fig. 4) also highlighted that the variability (SD) of inter-SE intervals is estrous cycle-dependent (Kruskal-Wallis: H(3) = 20.21, *P* = .0002) being the most variable (mean SD = 56.4 ± 4.9 minutes) in estrus and most consistent in diestrus (mean SD = 24.7 ± 2.9 minutes) (Supplementary File) (16).

**Figure 5.**
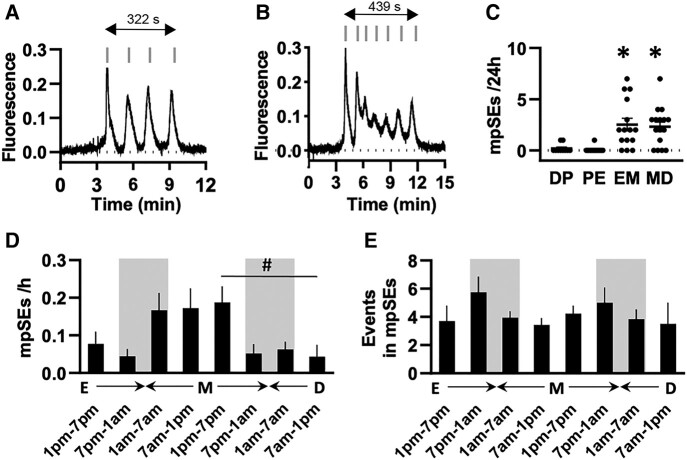
Features and distribution of multipeak synchronization events (mpSEs) exhibited by ARN^KISS^ neurons. (A-B) Representative GCaMP photometry traces showing mpSEs recorded in metestrus-estrus stages. The gray vertical lines above the traces point to the individual peaks of mpSEs that occur with SE intervals in <160-second intervals. Horizontal arrows with numbers show the time course of the mpSEs. (C) Scatter plot indicating the number of mpSEs over 24-hour recordings, during which mice were transitioning from diestrous to proestrous (DP, N = 10), proestrous to estrous (PE, N = 14), estrous to metestrous (EM, N = 15), and metestrous to diestrous (MD, N = 16) stages. (D-E) Bar graphs plotting the number of mpSEs per hour (D), and the average number of peaks per mpSEs (E) in 6-hour time blocks over 48 hours during transitioning from estrus across metestrus (M, N = 15) to diestrus (D, N = 16). The “lights off” period (from 7 Pm to 7 Am) is indicated by shading. All photometry recordings were started between 9 and 11 Am. Estrous stage was determined at the beginning and at the end of each recording. Each stage of the estrous cycle was defined as beginning at midnight. Data are represented by mean ± standard error of the mean. *P < .01 vs proestrus, and #*P* < .05 (Dunn multiple comparisons test).

We undertook continuous mode photometry to obtain high temporal resolution (10 Hz) profiles of mpSEs (Fig. 5A and 5B). This showed that an mpSE comprised multiple (usually 2-8, but up to 11) peaks in close succession to one another (<160-second interval). These multiple peaks could occur as multiple normal SEs (Fig. 5A) or, in other cases, there was no return to baseline between SEs, with mini-peaks occurring within 1 extended event (Fig. 5B).

The occurrence of mpSEs strongly depends on the estrous cycle (Kruskal Wallis H(3) = 24.74, *P* < .0001, Fig. 5C), arising almost exclusively in estrus and metestrus (∼2.5 and ∼2.3 per 24 hours, respectively). The vast majority of mpSEs emerge after midnight at the start of metestrus and remain through into metestrus (Fig. 5D and 5E). The number of peaks within an mpSE does not change across estrus and metestrus (Fig. 5E). All but 1 of the 18 mice (94%) exhibited mpSEs during estrus and/or metestrus. Very occasionally, mpSEs were observed in diestrus and proestrus (∼0.15 per 24 hours) representing ∼2% of all detected mpSEs across the dataset.

### K-means Clustering Captures the Dynamic Changes of ARN^KISS^ Neuron Population Activity

To establish a machine-learning based platform for assessing ARN^KISS^ neuron pulse generator activity, we performed unsupervised k-means clustering on parameters calculated from the population activity data of the ARN^KISS^ neurons recorded over 71 24-hour segments from 18 different mice (see Methods).

**Figure 6.**
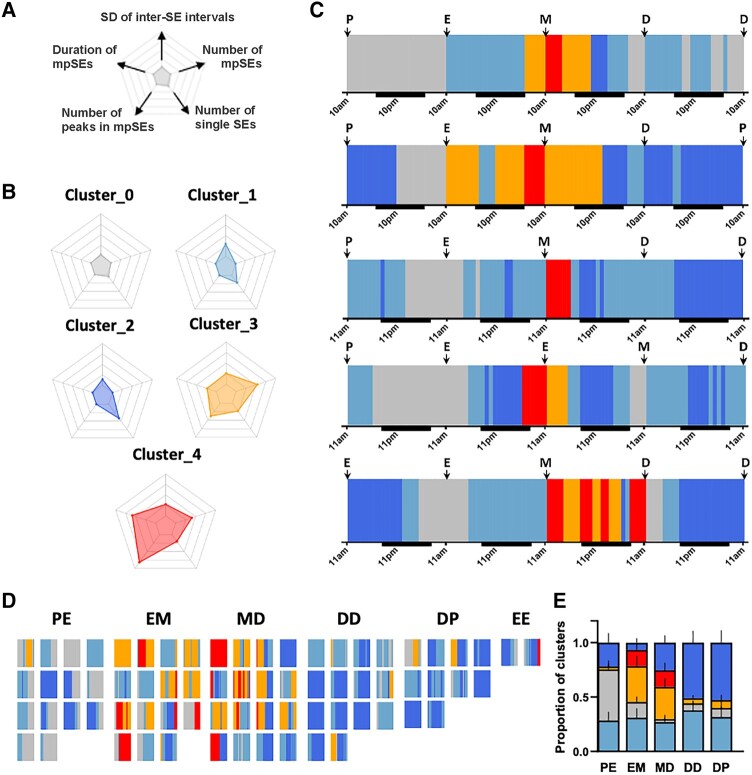
Unsupervised k-means clustering reveals dynamic changes in the activity profile of the ARN^KISS^ neurons with high temporal resolution. (A) Radar plot demonstrating parameters used for the cluster assignments with the following axes: (top) SD of inter-SE intervals, (top right) number of multipeak SEs (mpSEs), (bottom right) number of single SEs (with mpSEs counted as a single SE), (bottom left) number of peaks in mpSEs, (top left) duration of mpSEs. (B) Cluster centroid values for the normalized parameters used for k-means clustering. All axes have a minimum value of 0 and maximum value of 1. (C) Hourly k-means cluster assignments for 96-hour continuous photometry recordings from 5 mice. Colored fields represent the assigned clusters for the center (fourth hour) of the 7-hour time windows (see Methods). In each 24-hour segment of the recording, the first and the last 3 hours of each day were extrapolated from the assignment of the fourth and 21st hours, respectively. The exact time of vaginal lavage is indicated by an arrow (D, diestrus; E, estrus; M, metestrus; P, proestrus). Note that results of the vaginal cytology were used for later reference, not for the k-means clustering. (D) K-means cluster assignments for 24-hour segments of the whole dataset (N = 71) during transitioning from proestrus to estrus (PE, N = 14), estrus to metestrus (EM, N = 15), metestrus to diestrus (MD, N = 16), diestrus to proestrus (DP, N = 10) as well as during recordings when mice stayed in a stage for >24 hours, in diestrus (DD, N = 14) and estrus (EE, N = 2). (E) Bar graph indicating the proportion of the cluster assignments for each stage of the estrus cycle.

This clustering algorithm was able to assign all 1 242 7-hour time periods to just 5 different clusters (Fig. 6A and 6B) (Supplementary File) (16). Three clusters were assigned to times when regular SEs were observed (clusters 0, 1, 2) and 2 clusters to times when mpSEs were found (clusters 3, 4). The only exception was rare episodes of mpSE activity detected during the cluster_0 activity.

Cluster 0: regular SEs occurring with a low frequency (n = 207).

Cluster 1: regular SEs with a middle range of frequency and exhibiting the highest inter-SE variability (n = 408).

Cluster 2: regular SEs occurring quickly and with little inter-SE variation (n = 345).

Cluster 3: frequent, short-duration mpSEs (n = 193).

Cluster 4: less frequent, longer duration mpSEs (n = 89).

This approach identified distinct phases of ARN^KISS^ neuron activity across the estrous cycle (Fig. 6C and 6E). As expected, prolonged periods of cluster_0 activity occurred during the transition from proestrus into estrus, representing the slow and halted activity occurring after the LH surge. Following this, most animals moved to periods of intermingled cluster_1 and cluster_2 activity showing more frequent SE patterns, with the former (with higher SE variability) dominating. This was followed by interspersed cluster_3 and cluster_4 activity as periods of mpSEs dominated in metestrus before giving way again to primarily cluster_1 and cluster_2 patterns of behavior in diestrus. These patterns were observed in animals with 4-day continuous recordings (Fig. 6C) as well as the complete data set (Fig. 6D).

A clear relationship existed between cluster-type activity and stages of the estrous cycle (Fig. 6E). Although cluster_1 pattern with high inter-SE variability was most consistently observed across at all stages, cluster_0 dominated from proestrus-estrus and mpSE clusters_3 and _4 occurred primarily during the estrus to diestrus period (Fig. 6E). Nevertheless, it was evident that individual mice progressed through the cluster phases at very different rates (Fig. 6C) and that the patterns of the pulse generator activity are not always in perfect synchrony with the estrous stage. For example, two 4-day recorded mice stayed in estrus for 2 consecutive days with 1 (fourth plot on Fig. 6C) only showing cluster_0 activity associated with the first day of estrus, whereas the second mouse (fifth plot on Fig. 6C) only had cluster_0 activity on the second estrus day.

## Discussion

We report here a detailed assessment of ARN^KISS^ neuron synchronization activity across the mouse estrous cycle. This has identified a new pattern of pulse generator activity occurring predominantly during metestrus. We also document the impact of a mild stressor such as vaginal lavage on ARN^KISS^ neuron behavior. Using an automated machine-learning-based method, we further demonstrate significant inter-stage variability in pulse generator activity that is not necessarily coupled to the estrous cycle stage as determined by the vaginal cytology.

The present study involved 2 substantial methodological differences compared with previous ARN^KISS^ neuron GCaMP photometry studies (10, 11). First, we used a genetic mouse model in which a Cre-dependent, Tet-amplified cassette (13) drives GCaMP6 expression rather than the more traditional use of an adeno-associated virus (AAV) to deliver Cre-dependent GCaMP6. These “genetic” and “AAV-based” approaches result in ∼99% and ∼69% of ARN^KISS^ neurons expressing GCaMP6, respectively (10, 14). The genetic approach requires the breeding of a genetic cross but provides a more robust and consistent level of GCaMP expression in ARN^KISS^ neurons without the need for AAV infection of the brain.

The second difference came from our realization that long-term continuous recordings were possible with very little, if any, GCaMP bleaching when performed in the scheduled mode. This enabled us to extend our continuous recordings from typically 4 to 6 hours to 24 hours, and then up to 96 hours, and has provided high-resolution temporal data over the complete cycle. Consequently, we believe the present data set comes as close as practically possible to representing the real patterns of GnRH pulse generator activity in cycling mice.

### New Features of Pulse Generator Activity

In agreement with previous photometry (10) and pulse bleeding (17) studies, the average daily frequency of the pulse generator was relatively constant across metestrus, diestrus, and proestrus before slowing markedly on the evening of proestrus. The ability to now examine this activity continuously throughout each day of the cycle reveals several new features of pulse generator behavior. First, we note that despite pulse generator frequency and amplitude being rather stable across the entire follicular phase of the cycle, we have detected a transient drop in SE frequency in the morning of diestrus. The functional significance of this is unknown but it is interesting to note that estradiol levels peak at this time in mice (18).

Another feature is the curious stage-dependent association of lighting changes on pulse generator frequency. After “lights off,” SE frequency increased during estrus and metestrus, whereas after “lights on,” there was a decrease in SE frequency in diestrus and proestrus. Consistent with this, we previously noted that SE frequency did not change at “lights off” in diestrus and proestrus (10), whereas Goto and colleagues (11) found SE frequency to increase at “lights off” during estrus and metestrus, as well as diestrus. The lack of a “lights-on” effect in estrus may result from the already slow pulse generator activity occurring on estrus morning. It is possible that circadian inputs to the pulse generator may exist as both vasoactive intestinal peptide and vasopressin modulate the activity of ARN^KISS^ neurons (19). However, the potential mechanisms underlying how transitions between dark and light phase may impact the pulse generator remains unknown, as does their relationship to fluctuating gonadal steroid hormones.

The third new feature is that of mpSE behavior beginning during the estrus-metestrus transition and occasionally extending into diestrus. It is surprising that this striking pattern of activity in intact female mice was missed in prior investigations. This may have been due to the suboptimal GCaMP signal provided by AAV transduction and the use of short recordings in which the transient stress from photometry cable connection may not have passed. We detected mpSEs in 17 of 18 mice, suggesting that there is no specific subpopulation of ARN^KISS^ neurons that gives rise to these events. A mpSE comprises several SEs occurring within 160 seconds of each other in quick succession. These mpSEs share some similarities with ARN^KISS^ neuron SEs observed in gonadectomized mice where up to 4 SEs occur within ∼4 minutes of each other to generate tightly clustered episodes of activity (20, 21). The principal differences appear to be a much greater number of individual events clustered together in mpSEs and, because of their smaller amplitude, most but not all events completing before the next starts. The mechanisms behind this pattern of behavior are unknown. According to the “glutamate 2-transition” model for ARN^KISS^ neuron synchronization (14), we would speculate that there may be insufficient dynorphin tone at this time to restrain the continual emergent glutamate-AMPA mini-synchronizations from transcending to full SEs. Although the mpSEs occur several hours after the postovulatory progesterone surge, estradiol levels are relatively unchanged at these times (18), suggesting that unlike the gonadectomized state (21), estradiol may not be the primary factor determining mpSE behavior.

Whether the multiple peaks within an mpSE translate into multiple LH pulses is unknown. Because the LH pulse frequency of 1 per hour (17) is identical to the frequency of mpSE clusters during metestrus, it is extremely unlikely that an mpSE generates more than 1 LH pulse. However, cycle stages exhibiting mpSEs have average LH pulse amplitudes that trend higher than at other stages (17). Thus, it is possible that mpSEs generate higher amplitude LH pulses. Unfortunately, the currently available methods for determining the pulsatile LH secretion using tail-tip pulse bleeding with a 5-minute sample interval (10, 20, 22) do not permit a direct comparison between the LH secretory patterns and mpSEs, in which peaks occur every 45 to 160 seconds. We have previously found it nearly impossible to determine the profile of LH pulses generated by clustered SE activity in ovariectomized mice because this immediately reverts to singlet SE activity in response to the stress of tail-tip bleeding (21).

### Machine-learning Algorithm for Pulse Generator Activity Analysis

By applying unsupervised k-means cluster analysis, we show that the activity pattern of the pulse generator across the estrous cycle can be described automatically without labeling the activity data according to the vaginal cytology. Based on the previously calculated features, k-means clustering identified 5 separate clusters in the pulse generator activity, each of which had a unique, well-defined pattern of predictive features. Once defined, the combination of these clusters can describe the activity pattern of the pulse generator at each stage of the cycle. We found that the proportion of cluster_0 activity, characterized by low SE frequency, increased from metestrus to proestrus becoming dominant on proestrus evening, whereas the proportion of cluster_2 behavior with quick and regular SE pattern increased from the estrus to metestrus and diestrus to proestrus periods and prevailed in diestrus. As expected, mpSE behavior, represented by cluster_3 and cluster_4, was almost only found from estrus through to diestrus. Cluster_1 with its relatively slow and irregular pattern of activity, however, seems to be present in all stages at similar proportions and may represent a transition zone of behavior.

Importantly, k-means clustering revealed high within-stage variability in the activity pattern of the pulse generator that had not been detectable previously. The highest within-stage variability appeared during the estrus to metestrus and metestrus to diestrus recordings, whereas the rest of the estrous cycle showed relatively similar cluster composition. In addition, we note that there can be considerable variation in cluster composition and the duration of clusters between animals of the same stage. Although it might be predicted that the duration of cluster_0 activity is dependent on the level and duration of the periovulatory progesterone rise (10), it is unclear what factors may be regulating the duration of mpSE-like behavior.

One insight into the factors controlling pulse generator activity comes from the observation that vaginal lavage but not “sham” handling of trained mice results in a significant reduction in ARN^KISS^ neuron SE frequency. This highlights the importance of presumably modulatory stress or ascending sensory inputs on the behavior of the pulse generator, and several brain regions are known to provide afferents to the ARN^KISS^ neurons (23, 24). From a practical perspective, these observations indicate that the normal free-running pattern of pulse generator activity can be masked by experimental procedures for a certain amount of time depending on the procedure.

Finally, the cluster analysis reveals that pulse generator activity is not always in perfect synchrony or tempo with the estrous stage, as determined by single-point vaginal cytology. Whereas changes in vaginal cytology are driven by the variable estradiol levels across the cycle (25), the impact of fluctuating estradiol on pulse generator activity would appear to be much less substantial. Hence, it is possible that any lack of synchrony between pulse generator activity stage and the estrous cycle arises from variable modulatory effects of gonadal steroid-independent afferent inputs to the ARN^KISS^ neurons in each mouse (23, 24).

It is perhaps also important to note that the detailed tempo of estrous cycle progression as defined by vaginal cytology is unknown but may also be variable between mice. Notwithstanding these considerations, it is important to note the high level of synchrony between mice during the late proestrous to estrous stages where elevated progesterone is likely responsible for the robust and marked suppression of pulse generator activity.

## Summary

By using long-term uninterrupted GCaMP6 fiber photometry recordings, we provide a detailed assessment of ARN^KISS^ neuron pulse generator activity across the mouse estrous cycle. We reveal a new pattern of activity occurring mostly in metestrus and also highlight the impact of light changes as well as vaginal lavage on the activity pattern of the ARN^KISS^ neurons. Using k-means clustering, we further demonstrate significant inter- and within-stage variability in the pulse generator activity and stress that transitions between estrous cycle stages are highly variable between animals. Looking ahead, we believe that k-means clustering will be an important tool in describing the behavior of the pulse generator and aid in the understanding of its regulation by hormonal and afferent inputs.

## Data Availability

Datasets generated during and/or analyzed during the current study are publicly available from the University of Cambridge Apollo Repository (16).

## Abbreviations

AAV: adeno-associated virus
ARN: arcuate nucleus
ARN^KISS^: ARN kisspeptin
mpSE: multipeak synchronization event
RM: repeated measures
SE: synchronization event
